# Reach-to-grasp kinematics and kinetics with and without visual feedback in early-stage Alzheimer’s disease

**DOI:** 10.1186/s12984-022-01108-1

**Published:** 2022-11-10

**Authors:** Jianhong Zhang, Yunling Xiao, Zong-Ming Li, Na Wei, Leitong Lin, Ke Li

**Affiliations:** 1 School of Control Science and Engineering, Shandong University, Jinan, 250061 China; 2 Department of Geriatrics, Qilu Hospital, Shandong University, Jinan, 250012 China; 3 Department of Orthopaedic Surgery, University of Arizona, Tucson, AZ 85724 USA

**Keywords:** Alzheimer’s disease, Sensorimotor control, Reach-to-grasp performance, Kinematics, Kinetics

## Abstract

This study aimed to investigate the effects of early-stage Alzheimer’s disease (AD) on the reach-to-grasp kinematics and kinetics with and without visual supervision of the grasping arm and hand. Seventeen patients who had been diagnosed with early-stage AD and 17 age- and gender-matched, cognitive normal (CN) adults participated in the experiment. A mirror operating system was designed to block the visual feedback of their grasping hand and forearms but to virtually show grasped targets. The target for reach-to-grasp kinematics was a reflective marker installed on a base; and the target for reach-to-grasp kinetics was a custom-made apparatus installed with two six-component force/torque transducers. Kinematics and kinetic parameters were used to quantify the reach-to-grasp performances. Results showed that the early-stage AD remarkably decreased the reaching speed, reduced the grasping accuracy and increased the transportation variability for reach-to-grasp kinematics. For kinetic analysis, early-stage AD extended the preload duration, disturbed the grip and lift forces coordination, and increased the feedforward proportion in the grasping force control. The AD-related changes in the reach-to-grasp kinematic and kinetic parameters depended on visual feedback and were associated with nervous system function according to correlation analyses with the neuropsychological testing. These results suggest that the abnormal kinematic and kinetic characteristics may correlate with the neuropsychological status of early-stage AD, and that the reach-to-grasp kinematic and kinetic maneuver could potentially be used as a novel tool for non-invasive screening or evaluation of early-stage AD.

## Introduction

Alzheimer’s disease (AD) is one of the most prevalent dementia. More than 50 million people have been diagnosed with AD and the prevalence will increase twofold in the next few decades worldwide [1]. AD commonly manifests as cognitive decline, irreversible memory loss, disorientation and psychiatric symptoms. Effective management of AD relies on early screening and diagnosis, followed by proper interventions to delay its progression [2, 3]. The preliminary diagnosis of AD is made by a combination of clinical criteria which includes mental status tests, neurological examination and brain imaging [4]. For example, the frequently used clinical examinations relying on patient self-reports and clinician judgements have limitations in the objectivity and precision [5]. Evaluating β or protein tau from blood serum may serve as a biomarker for early-stage AD, but the sophisticated and invasive operation keeps it from being widely applied [6]. Hence, searching for objective, noninvasive, and practical biomarkers would still be an intriguing issue for promoting the early diagnosis and screening of AD.

There are increasing evidence showing that the neurodegeneration with AD involves changes of the sensory or motor functions. Abnormities in gait [7], lost of postural equilibrium [8], deficiency in language and skilled movement [1, 9] could be early signs for cognitive decline, and associated with dementing process. Sensorimotor markers are independent of culture background and educational levels, thereby would be suitable for clinical use. Although sensorimotor markers as an independent contributor to the cognitive decline remain controversial, growing evidence suggests that sensorimotor variables incorporating cognitive assessment would improve the evaluation of early cognitive decline [7].

The unique ability of human to interact with the environment lies in their skilled use of the hands for dexterous object manipulation [10]. Target-directed reaching involves localization of the target in space, transportation and orientation of hand, and re-shaping and coordination of the hand and digits relative to the target [11, 12]. Multimodal sensory including vision, haptics and proprioception may play a role in spatial and temporal regulation for the reach-to-grasp behavior. Visual information about the position and characteristics of the object may facilitate to form appropriate sequence of motor commands for specific manipulation goals [13, 14]. Effects of visual feedback on reach-to-grasp performance are manifest in direction dependence [15], selective perturbation of the target [16] and synchronized hallmarks of the sub-movements for coordination [17]. Models have also been developed to quantify the contributions of vision and proprioception in position estimation for motor planning [18]. In addition, tactile sensors in fingertips can detect the physical properties of the object including the curves and friction of the contact area and encode the information about the weight and center of mass of the object [19, 20].

A successful reach-to-grasp performance involves multimodal sensory information continuously and seamlessly integrated with motor commands and memory with feedback and feedforward mechanisms. The feedforward mechanism allows individuals to program the appropriate motor commands prior to reaching or grasping according to previous experiences; whereas the feedback mechanism adjusts the reaching and grasping according to real-time sensory information [21]. Kinematic and kinetic parameters (e.g. attitude and joint angles of the grasping hands, moving speed and trajectory of the target object, the magnitude and direction of fingertip forces and moments) have been examined for reach-to-grasp performance [22, 23]. The sensorimotor integration for reach-to-grasp kinematics and kinetics is under the government of center nervous systems (CNS). Superposition of visual and proprioceptive maps for accurate reaching resides in the posterior parietal cortex, and corticospinal drives to brachioradialis and anterior deltoid can be strongly excited during reaching, hand transportation and digit orientation [24]. Cognitive degenerations or lesions in CNS could disturb the central mechanism, thus potentially detectable from a reach-to-grasp performance. However, little is known about a functional decay of kinematics and kinetics of reach-to-grasp movement associated with early-stage AD.

This study aimed to investigate the effects of early-stage AD on the reach-to-grasp kinematics and kinetics with and without visual supervision of the grasping arm and hand. The reduced visual feedback of the grasping arm and hand may help explore AD-related changes in sensorimotor function. We hypothesized early-stage AD would affect the kinematic (e.g. accuracy and coordination of reaching) and kinetic (e.g. force and moment control) performance particularly without visual feedback on the grasping hand and forearm. We further hypothesized that the abnormal kinematic and kinetic characteristics would correlate with the status of early-stage AD.

## Materials and methods

### Subjects

Seventeen patients who had been diagnosed with early-stage AD (Age 64.9 ± 6.5 y, 7 male, 10 female) and 17 age- and gender-matched cognitive normal (CN, Age 64.9 ± 5.8 y, 7 male, 10 female) adults participated in the experiment. All subjects were right-handed with normal or corrected-to-normal vision. The handedness of each subject was based on their self-report followed by assessment of Edinburgh Handedness Inventory. The AD patients were recruited from the Department of Neurology at Qilu Hospital of Shandong Province, China. They were diagnosed as early stage of AD according to the criteria of National Institute of Neurological and Communicative Diseases and Stroke/Alzheimer's Disease and Related Disorders Association by professional therapists. The diagnosis and staging were based on comprehensive judgement according to neuropsychological tests, braining imaging, and amyloid-beta and tau in cerebrospinal fluid [2, 25]. Neuropsychological tests including the Mini–Mental State Examination (MMSE), the Montreal Cognitive Assessment (MoCA), the Hamilton Anxiety Scale (HAMA) and the Hamilton Depression Scale (HAMD) were performed on each AD patient. Inclusion criteria were: (1) over 50 years old; (2) clear mental state; and (3) ability to understand the instructions. Exclusion criteria were: (1) late-stage AD; (2) sever stroke; (3) Parkinson's disease; (4) history of upper-limb fractures or upper-limb diseases including but not limited to scapulohumeral periarthritis, scapular soreness, ulnar tunnel syndrome, radial tunnel syndrome, carpal tunnel syndrome, finger fractures, tenosynovitis, elbow ankylosis or peripheral neuropathies. Each subject was fully informed the purposes of this study and given informed consent prior to the experiment. The experimental procedures were approved by the Institutional Review Board of Shandong University (KYLL-2020(KS)-340) and were in accordance with the Declaration of Helsinki.

### Experimental setup

**Fig. 1:**
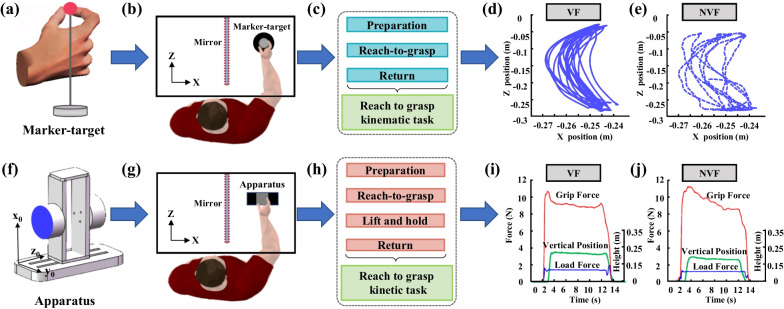
Experimental set-up, test protocol and representative signals of the reach-to-grasp performance. **a** A spherical retro-reflective marker placed a custom stand as the target for the reach-to-grasp kinematic task. **b** The mirror system for reach-to-grasp kinematic test. The reflective side of the mirror was facing the left alley and the grasping hand was behand the mirror in the right alley. **c** Protocol of reach-to-grasp kinematic test. **d**, **e** Trajectories in the horizontal plane (x–z plane) during reaching, grasping and returning under VF (**d**) and NVF (**e**) conditions from a representative AD patient. **f** The apparatus for reach-to-grasp kinetics. **g** The mirror system for reach-to-grasp kinetic test. **h** Protocol of reach-to-grasp kinetic test. **i**, **j** Grip force, load force and vertical position of the apparatus during precision grip under VF (**i**) and NVF(**j**) conditions from a representative AD patient

Retro-reflective markers were affixed to the dorsal surface of the right hand of each subject. The markers included nail marker-clusters on distal segments of the thumb and index finger [26, 27], hand marker-cluster along the second metacarpal, and single marker proximal to wrist. An optical three-dimensional (3D) motion capture system (OptiTrack™, USA) was used to track the position of the markers. A reflective marker installed on a base (Fig. 1a) was used as the grasping target for kinematic task. A custom-made apparatus installed with two six-component force/torque transducers (Nano 17, ATI Industrial Automation, Inc., Apex, NC) was used as the grasping target for the kinetic task (Fig. 1f). The transducers were mounted on the apparatus by precisely positioning that the *x*-axis and *y*-axis were along the vertical and horizontal directions in the contact surface of each transducer, and the *z*-axis was in the perpendicular direction to the contact surface (Fig. 1f). The grip surfaces with a span of 50 mm were covered with 100-grit sandpaper to increase the coefficient of friction (Fig. 1f). The gross weight of the instrumented apparatus was 172 g. Data was collected using a custom LabVIEW program (National Instrument, Austin, TX). Force signals were amplified and multiplexed using an ATI interface boxes (ATI Industrial Automation, Inc., Apex, NC), converged to 16-bit analog–digital converters (PCIe-6343, National Instrument, Austin, TX) and collected at a sampling frequency of 1000 Hz.

A mirror operating system was designed to block the visual feedback of the grasping hand and forearms from reach-to-grasp action [23]. The mirror was approximately 50 cm × 49 cm height and width, and 1 cm in thickness. After the mirror was in place, the space in front of the subject could be divided into two alleys. The reflective and the coating sides of the mirror are facing the left and right alleys, respectively. A marker-target for the kinematic test or a surrogate apparatus for the kinetic test was placed on the left alley, so that at the symmetric position with respect to the mirror a target or an apparatus for grasping was observable. Using this mirror system, the subject’s reaching right hand was behind the mirror in the right alley so that the visual supervision of the grasping hand and forearm could be blocked but the visual information about the target’s location was remained, which was designated as the without visual feedback (NVF) condition (Fig. 1b, g). By contrast, once the mirror system was removed, all the visual information about the target and the grasping hand and arm were available, which refers to the visual feedback (VF) condition. For one who completed the tests under the two visual conditions, the differences between the VF and NVF conditions should be mainly attributed to the effects of vision, rather than the other factors such as the differences in muscle strength between subjects. Comparison between the two visual conditions could allow to observe the AD-related sensorimotor deficits.

### Experimental procedures

The subjects sat comfortably at a table, with the right elbow flexed approximately 90° in the parasagittal plane, the left hand naturally on the left side of body. The grasping target was rigidly fixed on the testing table, aligned with the subject’s right shoulder and at a distance of 35 cm in front of the subject. The right hand was placed on the start position of the table before each trial.

For each kinematic trial, the subject was required to reach and grasp the marker-target with the tips of the thumb and index finger following auditory cues for consecutive five times. After receiving an audible ‘go’ command, the subject reached with his or her right hand towards the virtual target. To minimize dwell near contact, the subject immediately returned the hand to the starting position on the third beep to complete the trial. The subject was instructed to pinch the target with the thumb and index finger as accurately and consistently as possible (Fig. 1c). For each kinetic trial, the subject was instructed to reach and grasp the apparatus with his or her thumb and index finger. After receiving an auditory cue, the subject lifted the apparatus vertically about 13 cm above the testing table, and maintained the apparatus in the air as stably as he or she could for 5 s. After receiving another auditory cue, the subject replaced the apparatus at the testing table and then returned his or her grasping hand to the initial position (Fig. 1h). The reach-to-grasp kinematic and kinetic tests were performed equally in both the VF and NVF conditions.

### Data analysis

#### Reach-to-grasp kinematic metrics

All the kinematic signals recorded by the motion capture system were filtered with a fifth-order Butterworth digital filter at a cutoff frequency of 5 Hz. The onset of the reaching was determined once the velocity of the moving hand exceeded 5 mm/s. We defined the grasping time as the duration from the onset of reaching to the timepoint when the hand returned to the initial position (Fig. 1d, e).

**Fig. 2:**
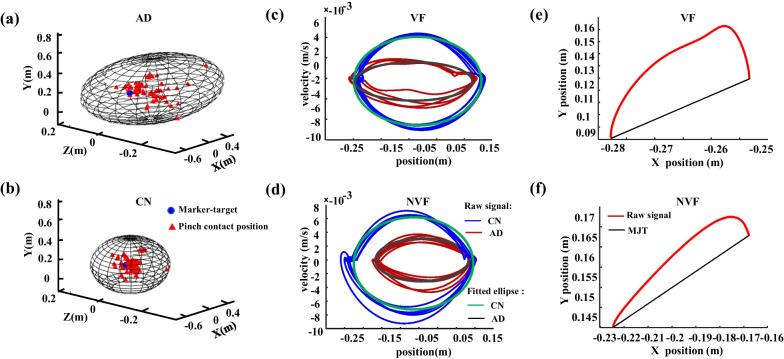
The reach-to-grasp kinematic performances of representative AD and CN subjects with different visual conditions. Distributions of the contact points by the thumb and index finger from a representative AD (**a**) and an age- and gender-matched CN (**b**) in NVF. The ellipsoids are fitting spheres including 95% of contact points. The volumes of the ellipsoids indicate the precision of grasping with respect to the target. Trajectories in velocity-position phase diagram during reaching, grasping and returning from a representative AD and a CN subjects with VF (**c**) and NVF (**d**). The minimum-jerk and actual trajectories from a representative AD during reaching in VF (**e**) and NVF (**f**) conditions

The spatial localizations of the contact points by the thumb and index finger for each subject were fitted by an ellipsoid, which included 95% of the pinch contact points by a principal component analysis (Fig. 2a, b). The volume (*Vol*) of the ellipsoid was computed as an estimation pinch accuracy *i*. A mean absolute error, defined as the Euclidean distance between the pinch contact location and the target, was calculated for each trial as follows:

1 $$MAE = \sqrt {(x_{t} - x_{0} )^{2} + (y_{t} - y_{0} )^{2} + (z_{t} - z_{0} )^{2} }$$

where $x_{t}$, $y_{t}$ and $z_{t}$ are coordinates of the pinch contact positions and the $x_{0}$,$y_{0}$ and $z_{0}$ are the coordinates of the target. The pinch contact location was determined following the method developed in a previous study [28]. Specifically, using each nail marker-cluster as a reference for a 3-D coordinate system, a spherical model of the respective digit finger-pad was represented. A virtual ‘‘nail-point’’ was computed as a projection along the cluster stem to the dorsal surface of the nail and served as the center of the respective sphere. Using digital calipers, each digit thickness was measured as the transverse distance from dorsal surface to digit-pad prominence of the distal segment and served as the sphere radius. A pinch contact between the thumb and index finger onto the target was assumed to occur according to two criteria: (1) the surfaces of the representative spheres for the two digits were separated by a distance equal to or less than 10 mm (i.e., the diameter of the marker target), and (2) the inter-distance velocity between the sphere centers was less than 15 mm/s. The distance between digit sphere surfaces is denoted as “inter-pad” distance. In addition, a mean absolute error, defined as the Euclidean distance between the pinch contact location and the target.

A movement harmonicity was proposed to quantify the movement variability of reach-to-grasp kinematics. Previous studies have demonstrated that the movement trajectories during a self-paced reach-to-grasp performance normally presents as elliptic curves in a velocity-position phase diagram (the *x*-axis is distance between the reaching hand and the target and the *y*-axis is the velocity of the hand, Fig. 2c, d). The movement harmonicity can be computed as follows:

2 $$\begin{gathered} \left\{ {\begin{array}{*{20}l} {MH = \frac{{|R_{ideal} - R_{measure} |}}{{R_{ideal} }}} \\ {R_{ideal} = \frac{{C_{ideal} }}{{A_{ideal} }}} \\ {R_{measure} = \frac{{C_{measure} }}{{A_{measure} }}} \\ \end{array} } \right. \hfill \\ \hfill \\ \end{gathered}$$

where the $C_{ideal}$ and $A_{ideal}$ are the circumference and area of an ideal ellipse whose major axis equals to the distance between the initial hand position and the target, and minor axis equals to the maximum velocity in the velocity-position phase diagram; the $C_{measure}$ and $A_{measure}$ are the circumference and area of the fitting ellipse of the movement trajectories in the velocity-position phase diagram.

A mathematic model [29] based on theory of dynamic optimization [30] was applied to quantify motor coordination during reach-to-grasp maneuver. Briefly, an objective function for motor coordination can be defined as follows:

3 $$\mathop {\arg \min }\limits_{x(t),y(t)} (\frac{1}{2}\int_{0}^{{t_{f} }} {\left[ {\left( {\frac{{d^{3} x(t)}}{{dt^{3} }}} \right)^{2} + \left( {\frac{{d^{3} y(t)}}{{dt^{3} }}} \right)^{2} } \right]} \, dt)$$

where $x(t)$ and $y(t)$ are the real-time coordinates of the hand in a planar motion, $t_{f}$ is the movement duration. A minimum-jerk trajectory algorithm was applied to estimate $x(t)$ and $y(t)$ that minimize the function [3]. The $x(t)$ and $y(t)$ can be expressed as fifth order polynomials as follows:

4 $$\left\{ {\begin{array}{*{20}c} {x(t) = x^{s} + (x^{s} - x^{f} )( - 10(\frac{t}{{t_{f} }})^{3} + 15(\frac{t}{{t_{f} }})^{4} - 6(\frac{t}{{t_{f} }})^{5} )} \\ {y(t) = y^{s} + (y^{s} - y^{f} )( - 10(\frac{t}{{t_{f} }})^{3} + 15(\frac{t}{{t_{f} }})^{4} - 6(\frac{t}{{t_{f} }})^{5} )} \\ \end{array} } \right.$$

where the $(x^{s} ,y^{s} )$ and $(x^{f} ,y^{f} )$ are the initial and final coordinates of the reaching hand. The area between the trajectory of reaching hand and the curve formed by the (*x(t), y(t)*) in Eq. (4) of each trial serves as an indicator for motion coordination (Fig. 2e, f).

#### Reach-to-grasp kinetic metrics

**Fig. 3:**
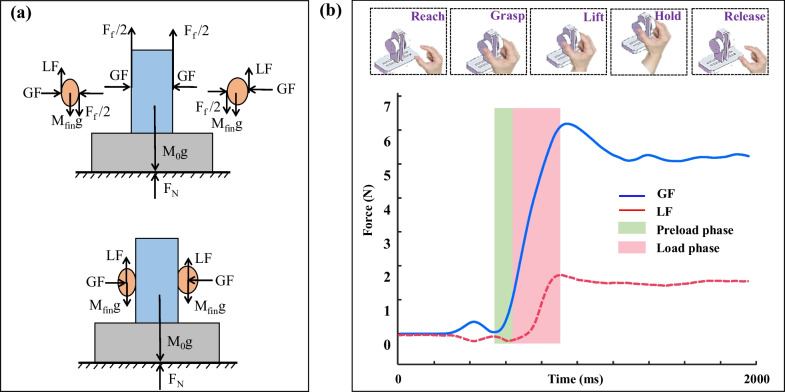
Force analysis for reach-to-grasp kinetic performance. **a** The force components applied by the thumb and index finger upon the apparatus; **b** the phases of reach-to-grasp kinetics and the *GF* and *LF* curves during grasping, lifting and holding the apparatus. The lifting phases can be further divided into the preload phase (in green) and the load phase (in pink)

The apparatus was used to measure the forces (*F*_*x*_, *F*_*y*_ and *F*_*z*_) and torques (*T*_*x*_, *T*_*y*_ and *T*_*z*_) of the thumb and index finger, separately. All force and torque components were recorded simultaneously and then filtered using a fifth-order Butterworth low-pass filter with a cutoff frequency at 30 Hz (Fig. 1i, j). The grip force, *GF*, applied by the thumb and index finger, were the average of the two perpendicular forces. The load force, *LF*, was the summation of the vertical lifting forces applied by the thumb and index finger (Fig. 3a). Reach-to-grasp kinetics can be generally divided into five phases, including to reach, grasp, lift, hold and release the apparatus (Fig. 3b). The lifting phase can be further divided into a preload and a load subphases. The preload phase (*T*_*pre*_) refers to the period from the moment when index finger and thumb first touched the object (the *GF* first exceeded 0.1 N for more than 2 s) to the onset of the load phase (the *LF* first exceeded 0.1 N) [31]. The load phase (*T*_*load*_) refers to the onset of the load phase to the moment when the load force overcame the gravity so that the object started to move (Fig. 3b).

**Fig. 4:**
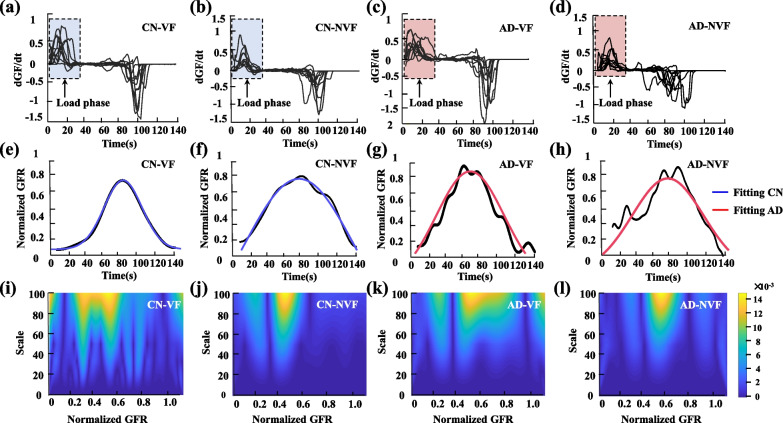
Grip force rate analysis for reach-to-grasp kinetic performance. The grip force rates of representative subjects, including a CN subject in VF (**a**) and NVF (**b**) conditions, and an AD patient in VF (**c**) and NVF (**d**) conditions. Normalized grip force rates (GFR) and their fitted Gaussian functions for the CN subject in VF (**e**) and NVF (**f**) conditions and AD patient in VF (**g**) and NVF (**h**). The time–frequency spectrogram of the normalized *GFR* with continuous wavelet analysis for the CN subject in VF (**i**) and NVF (**j**) and the AD patient in VF (**k**) and NVF (**l**)

The first derivative of *GF* versus time during the load phase was computed as grip force rate (*GFR,* Fig. 4a–d). A Gaussian function was used to fit the curve of *GFR* (Fig. 4e–h)*,* and the root mean square errors (*RMSEs*) between the normalized *GFR* and the fitted Gaussian curve were calculated to quantify their differences. A continuous wavelet transform (*CWT*) with slow and fast bell-shaped functions (Mexican Hat waveform) was used to examine the time–frequency characteristics of the normalized *GFR* (Fig. 4i–l). The slow bell-shaped function indicates the components with lower frequency (or higher scale), reflecting the slowly changed *GFR* components. By contrast, the fast bell-shaped function indicates the components with higher frequency (or lower scale), which reflects the fast changes in *GFR*. To simplify the calculation, the slow bell-shaped component $S(b)$ was defined as the average of the 5 scales of the slow bell-shaped function in formula (5). Similarly, the fast bell-shaped component $F(b)$ was defined as the average of the 5 scales of the fast bell-shaped function. The percentage ratio $R(b)$ was calculated as the division of the slow bell-shaped component to the sum of slow and fast bell-shaped components as specified in formula (5).

5 $$\left\{ {\begin{array}{*{20}l} {Slow:S(b) = \frac{1}{5}\sum\limits_{i = 1}^{5} {CWT(a_{i} ,b)} } \\ {Fast:F(b) = \frac{1}{5}\sum\limits_{j = 1}^{5} {CWT(\tilde{a}_{j} ,b)} } \\ {R(b) = \frac{S(b)}{{S(b) + F(b)}} \times 100\% } \\ \end{array} } \right.$$

where $a_{i}$ = 15, 17.5, 20, 22.5, and 25 for the slow components and $\tilde{a}_{j}$ = 70, 80, 90, 100, and 110 for the fast components. The average of $R(b)$ during the load phase was calculated as a parameter for the statistical analysis.

**Fig. 5:**
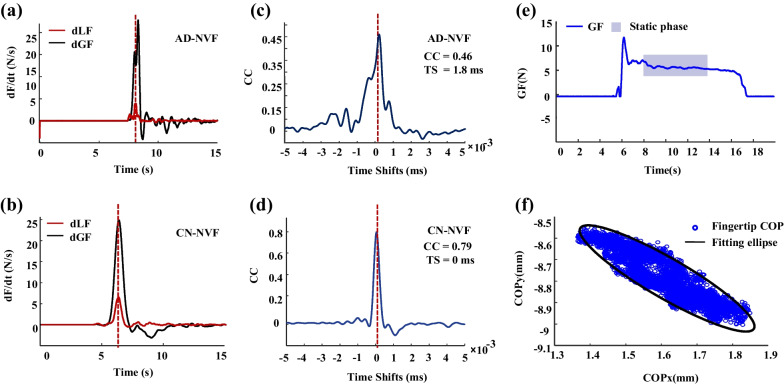
The *GF-LF* coordination and the center of pressure areas. The deviations of GF and LF of representative subjects with NVF for AD (**a**) and CN (**b**). The cross-correlation analysis based on the GF (**c**) and LF (**d**) rates of change. The maximal coefficient of correlation (CC) and the time shifts (TS) were used to quantify the GF and LF coupling. The coefficient of variation defined as the ratio of the standard deviation to the mean of *GF* of the hold phase (**e**). Distributions of the fingertip center of pressure and its area estimated by a fitted ellipse (**f**)

The *GF*-*LF* coordination was estimated by computing a cross-correlation function based on the rates of change of the *GF* and *LF*. For each trial, the maximal coefficient of correlation (*CC*) and the time shifts (*TS*) were used to quantify the *GF* and *LF* coupling (Fig. 5a–d). The coefficient of variation (*COV*) which was defined as the ratio of the standard deviation of *GF* to the mean of *GF* during the first 5 s of the hold phase was used to quantify the variation of pinch force control (Fig. 5e). To determine the thumb and index finger tip positions on the manipulandum, the* x* and *y* coordinates of the center of pressure (*COP*) of each fingertip were measured during the hold phase. The *COP* data were fitted by an ellipse for the thumb and index finger (Fig. 5f), separately. The area of the ellipses in which 95% of the *COP* were located was computed as an estimate of the *COP* variability.

The validity of reach-to-grasp kinetic and kinematic parameters were examined with neuropsychological tests. Correlations analyses between the reach-to-grasp parameters and the scores of MMSE, MoCA, HAMA and the HAMD were performed for the AD group. The correlations were analyzed between each kinematic or kinetic parameters and each neuropsychological test scores, individually without consideration of multiple comparison. Only the correlations fulfilling statistically significance were retained as meaningful results.

### Statistical analysis

All statistical analyses were performed using SPSS 25.0 (SPSS Inc., Chicago, IL). The kinematic and kinetic parameters were firstly examined for normality using a Kolmogorov–Smirnov test (K-S test). Analysis of variance (ANOVA) with repeated measures were employed to examine the differences of kinematic and kinetic parameters between the AD and CN groups as the between-subject factor across and the VF versus NVF conditions as the within-subject factor. Independent samples *t*-tests were applied to examine the difference in the kinematic and kinetic parameters between the AD and CN groups. Paired samples *t*-tests were applied to examine the effects of visual feedback for both the AD and CN groups. Correlation analyses between the neuropsychological test scores, including the MMSE, MoCA, HAMA, and HAMD, and the kinematic or kinetic parameters were further performed. A *p*-value of less than 0.05 was considered statistically significant.

## Results

### Results of reach-to-grasp kinematics

**Fig. 6:**
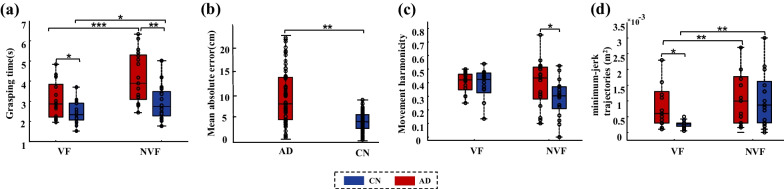
Reach-to-grasp kinematics for the AD and CN groups in VF and NVF. **a** Grasping time; **b** mean absolute error; **c** Movement harmonicity; **d** Minimum-jerk trajectories. *p < 0.05; **p < 0.01; ***p < 0.001

The grasping time for the VF and NVF conditions during the reach-to-grasp kinematic task are shown in Fig. 6a. The ANOVA tests showed significant main effects of AD (*F*_(1,32)_ = 11.477, *p* < 0.01) and visual conditions (*F*_(1,32)_ = 26.777, *p* < 0.001) on the grasping time. Specifically, in the VF condition, the grasping time were 2.42 ± 0.53 s for CN and 3.07 ± 0.87 s for AD (*t* = − 2.512, *p* < 0.05); in the NVF condition, the grasping time were 2.93 ± 0.83 s for CN and 4.17 ± 1.23 s for AD (*t* = − 3.428, *p* < 0.01). Compared with the VF conditions, relatively higher grasping time was found in the NVF for both the AD (*t* = **− **5.038, *p* < 0.001) and CN (*t* = **− **2.240 *p* < 0.05) groups. The distribution of grasping contact locations and its fitting ellipsoid in the NVF condition are demonstrated in Fig. 2a and b. The AD patients showed a larger volume of the fitting ellipsoid than the CN subjects (0.0933 m^3^ for AD vs. 0.0266 m^3^ for CN). The mean absolute error of the AD patients were significantly higher than those of the CN group in the NVF condition (*t* = 8.728, *p* < 0.01, Fig. 6b). Results of the movement harmonicity and minimum-jerk trajectory are shown in Fig. 6c and d, respectively. Repeated measures ANOVA showed significant main effects of group (AD vs. CN) on both the movement harmonicity (*F*_(1,32)_ = 4.239 *p* < 0.05) and minimum-jerk trajectory (*F*_(1,32)_ = 5.822, *p* < 0.05).The movement harmonicities of AD were significantly higher than those of the CN group in NVF (*t* = − 2.828, *p* < 0.05). No effects of visual conditions (*p* = 0.310) or the visual × group interaction (*p* = 0.116) were found for the movement harmonicity. By contrast, significant differences was found between the VF and NVF conditions for the minimum-jerk trajectory (*F*_(1,32)_ = 9.375, *p* < 0.01); but no significant interaction between the group and visual conditions was observed (*p* = 0.097). Compared with the VF conditions, relatively higher minimum-jerk trajectories were found for both the AD (*t* = − 3.335, *p* < 0.01) and CN (*t* = − 4.101, *p* < 0.01) groups under the NVF condition.

### Results of reach-to-grasp kinetics

**Fig. 7:**
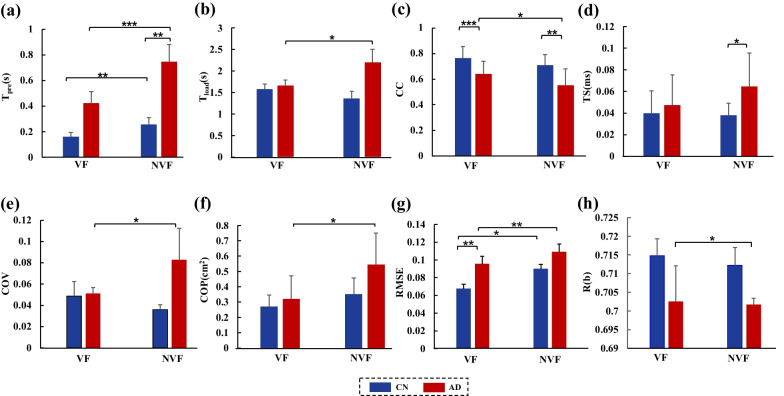
Reach-to-grasp kinetics for both AD and CN groups in VF and NVF. **a** Duration of preload phase; **b** duration of load phase; **c** maximal coefficient of correlation; **d** time shifts; **e** coefficient of variation; **f** center of pressure areas; **g** root mean square error; **h** *R(b)* of grip force rate. *p < 0.05; **p < 0.01; ***p < 0.001

Results of the *T*_*pre*_ and *T*_*load*_ during the grasping kinetic task are shown in Fig. 7a and Fig. 7b, respectively. The repeated measures ANOVA showed significant main effects of group (*F*_(1,32)_ = 10.152, *p* < 0.001) and visual conditions (*F*_(1,32)_ = 47.620, *p* < 0.01) on the *T*_*pre*_, with significant interaction observed between the group and visual conditions (*F*_(1,32)_ = 5.191, *p* < 0.05). Relatively higher *T*_*pre*_ was found in NVF than in VF for both the AD (*t* = − 8.922, *p* < 0.001) and CN (*t* = − 2.979, *p* < 0.01) groups. In VF, no significant difference was found in the *T*_*pre*_ values between groups (*p* = 0.095); in NVF, the *T*_*pre*_ values of AD were significantly longer than those of CN (*t* = 3.090, *p* < 0.01). The repeated measures ANOVA showed significant main effects of visual conditions on the *T*_*load*_ (*F*_(1,32)_ = 6.807, *p* < 0.05). No significant difference was found between the AD and CN groups for the *T*_*load*_ (*p* = 0.664).

Results of the *CC* and *TS* are shown in Fig. 7c and d, respectively. Repeated measures ANOVA showed significant differences of *CC* between the AD and CN groups (*F*_(1,32)_ = 31.172, *p* < 0.001). Significant differences was found on visual condition (*F*_(1,32)_ = 7.962, *p* < 0.01), without significant interactions between group and visual condition (*p* = 0.478). The AD showed lower *CC* values than the CN with VF (*t* = − 4.067, *p* < 0.001) and NVF (*t* = − 3.856, *p* < 0.01). There was significant difference in *TS* between the AD and CN groups (F_(1,32)_ = 10.359, *p* < 0.001). The AD showed relatively higher *TS* than the CN in NVF (*t* = 2.409, *p* < 0.05**)**. No significant interaction was found between group and visual conditions for *TS* (*p* = 0.613).

The *COV* and *COP* are demonstrated in Fig. 7e and f, respectively. Repeated measures ANOVA showed significant main effects of visual conditions for the *COV* (*F*_(1,32)_ = 9.415, *p* < 0.01) and *COP (F*_(1,32)_ = 5.573, *p* < 0.05). No significant difference was found between the VF and NVF conditions for either the *COV* (*p* = 0.786) or *COP* (*p* = 0.410). For the AD groups, the *COV* and *COP* in NVF were significantly greater than those in VF (*COV*: *t* = − 3.636, *p* < 0.01; COP: *t* = − 2.500, *p* < 0.05). For the CN, however, no significant difference between the VF and NVF conditions was found for the *COV* (*p* = 0.252) or *COP* (*p* = 0.287).

The time course of normalized *GFR* and the fitting curves with Gaussian functions for the grasping kinetic task are shown in Fig. 7g, respectively. The repeated measures ANOVA showed significant main effects of groups (*F*_(1,32)_ = 10.303, *p* < 0.01) and visual conditions (*F*_(1,32)_ = 21.764, *p* < 0.001) on the *RMSE*. The AD showed significantly higher *RMSE* values than CN only in VF condition (*t* = 3.279, *p* < 0.01). The *RMSE* of AD with VF (0.090) was significantly lower than with NVF (0.110, *t* = − 4.074, *p* < 0.01); and the *RMSE* of the CN with VF was significantly lower than with NVF (*t* = − 2.922, *p* < 0.05). No significant visual × group interaction was found in *RMSE* (*p* = 0.811).

No significant difference was found in *R(b)* between the AD and CN groups (*p* = 0.785) (Fig. 7h). Visual conditions could affect the *R(b)* (*F*_*(*1,32)_ = 4.132, *p* < 0.05). For the AD group, the *R(b)* values with VF were significantly higher than that with NVF (*t* = 2.495, *p* < 0.05). No significant difference was found between the two visual conditions for the CN group (*p* = 0.407).

### Results of correlations between reach-to-grasp parameters and neuropsychological tests

**Fig. 8:**
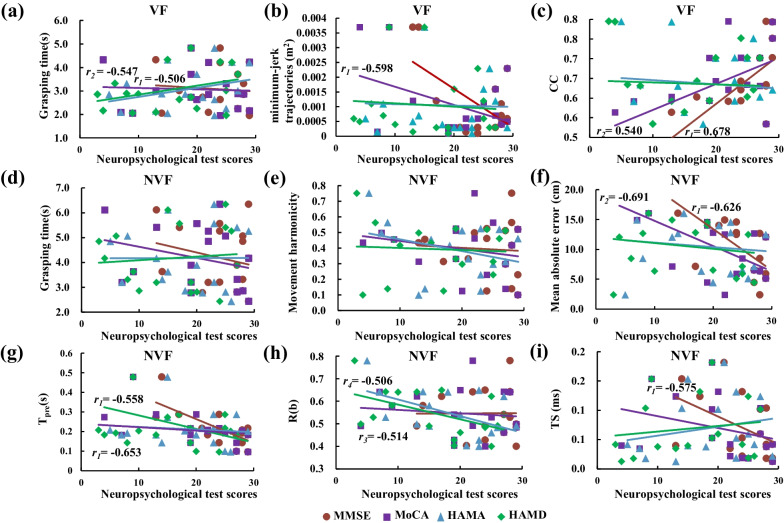
Correlations between the reach-to grasp parameters and the neuropsychological tests. **a**–**d** Results of VF condition. Correlations between the grasping time (**a**), minimum-jerk trajectories (**b**), the maximal coefficient of correlation (**c**), grasping time (**d**) and the MMSE, MoCA, HAMA, HAMD scores with VF. **e–i** Results of NVF condition. Correlations between the movement harmonicity (**d**), mean absolute error (**f**), the preload phase (**g**), R*(b)* (**h**), time shifts (**i**) and the MMSE, MoCA, HAMA, HAMD scores with NVF

The neuropsychological tests showed the MMSE, MoCA, HAMA, HAMD scores for the AD patients were 24.2 ± 5.2, 20.2 ± 7.7, 18.4 ± 7.2, and 15.8 ± 8.1, respectively. With VF, the MMSE was negatively correlated with the grasping time (*r*_*1*_ = − 0.506; *p* < 0.05, Fig. 8a) and the minimum-jerk trajectory (*r*_*1*_ = − 0.598; *p* < 0.05, Fig. 8b), and the MMSE was positive correlated with the *CC* (*r*_*1*_ = 0.678; *p* < 0.01, Fig. 8c). The MoCA was correlated with the grasping time (*r*_*2*_ = − 0.547, *p* < 0.05, Fig. 8a) and *CC* (*r*_*2*_ = 0.540, *p* < 0.05, Fig. 8c). With NVF, no similar correlation was observed between the grasping time and neuropsychological tests (Fig. 8d), or between the movement harmonicity and neuropsychological tests (Fig. 8e). The mean absolute error showed negative correlations with the MMSE (*r*_*1*_ = − 0.691; *p* < 0.01, Fig. 8f) and MoCA (*r*_*2*_ = − 0.626, *p* < 0.01, Fig. 8f). The *T*_*pre*_ was negatively correlated with the MMSE (*r*_*1*_ = − 0.653; *p* < 0.01, Fig. 8g) and MoCA (*r*_*2*_ = − 0.558; *p* < 0.05, Fig. 8g). *R(b)* was negatively correlated with the HAMA (*r*_*3*_ = − 0.514; *p* < 0.05, Fig. 8h) and HAMD (*r*_*4*_ = − 0.506; *p* < 0.05, Fig. 8h). In addition, the *TS* was correlated with the MMSE (*r*_*1*_ = − 0.575; *p* < 0.05, Fig. 8i).

## Discussion

This study aimed to investigate the effects of early-stage AD on the reach-to-grasp kinematics and kinetics. Two visual conditions (VF vs. NVF) were provided, in order to examine the AD-related changes under sensory modulation. Results showed that early-stage AD could remarkably decrease the reaching speed (e.g. increased grasping time), reduce the grasping accuracy (e.g. greater mean absolute error) and augment the transportation variability (e.g. increased movement harmonicity and minimum-jerk trajectory) for reach-to-grasp kinematics. In addition, the early-stage AD extended the preload duration (e.g. increased *T*_*pre*_), disturbed the *GF*-*LF* coordination (e.g. decreased *CC* and increased *TS*), and increased the feedforward proportion in the grasping force control (e.g. higher RMSE and lower *R(b)*). It is noteworthy that most of the AD-related changes highly relied on the visual conditions. Specifically, the grasping errors (mean absolute error), transportation variability (movement harmonicity), preload duration (*T*_*pre*_) and time shifts of the *GF*-*LF* coordination (*TS*) showed significantly higher values in AD than in CN under the NVF rather than the VF condition (Figs. 6, 7). The AD-related changes in the grasping kinematic and kinetic parameters were associated with nervous system function, which could be demonstrated from the moderate to strong correlations of the reach-to-grasp parameters with the MMSE, MoCA, HAMA or HAMD scores in AD (Fig. 8) [32].

The decreased reaching speed (increased grasping time) associated with early-stage AD suggests more time to initiate and execute goal-directed reaching movement. Previous studies found that the risk for cognitive impairment could be associated with slower gait in locomotion [33, 34] and slower initiation and execution of goal-directed pro-tapping task [35]. Individuals with early-stage AD demonstrated slower, clumsy, uncoordinated, and inconsistent handwriting movements than the healthy subjects [36, 37]. Considering the motion speed is associated with the coordination of multiple joints, evidence from the grasping time suggests that early-stage AD might lead to deficits or difficulties in coordination of multiple joints while executing reach-to-grasp task [38]. Reduced structural and functional integrity of prefrontal cortex and hippocampus, or dysfunction of the basal ganglia could be potential reasons for the slower actions [39]. In addition, motor planning and execution are considered to be related to the interconnection of multiple cortical regions [40, 41], and increased grasping time may also reflect a potential linkage between the systemic disorders across cortical regions and the behavioral manifestation [42].

The decreased grasping accuracy reflected by the greater mean absolute error in early-stage AD than in CN suggests that the neurodegeneration associated with the AD would impair the fine motor control for grasping kinematics. This changes could be resultant from the decreased localization associated with AD specifically when the visual feedback for the grasping hand was blocked [3]. Several cortical areas, such as Brodmann area 5 of the superior parietal lobe, the parieto-occipital junction and the premotor areas, may play a role in positioning or localizing an object in a 3D space [43]. The structural or functional changes in these cortical areas due to AD potentially lead to the decreased grasping accuracy. In addition, the poor spatial localization performance observed in the early-stage AD may be associated with the trans-neuronal spread of pathological tau within the entorhinal cortex-hippocampal circuit [44]. Accumulation of amyloid-β pathology in the retro splenial cortex associated with AD may also attribute to the decreased grasping accuracy [45].

The higher movement harmonicity and higher minimum-jerk trajectory describe transportation variability during reach to grasp an object. The movement harmonicity is an indicator of movement harmonicity. The values of movement harmonicity closer to 0 indicate more harmonic movements [46]. Minimum-jerk trajectory describes ideal trajectories potentially existing in any target-oriented hand motions according to the minimum-jerk principle. The higher minimum-jerk trajectory implies augmented deviations between the hand transportation to the minimum-jerk trajectory [29]. Results of movement harmonicity and minimum-jerk trajectory confirm our hypothesis that the neurodegeneration associated with AD may remarkably increase the movement variability for hand transportation during reach-to-grasp an object, suggesting potentially altered central or peripheral neuroregulatory control in early-stage AD .

### Grasping kinetic metrics

The increased *T*_*pre*_ associated with AD suggests a longer transition from the kinematic control for reaching to kinetic control for precision grip. The increased *T*_*pre*_ in AD was probably due to the difficulty increased with degraded neural function in switching the subgoals of a consecutive motor program, resulting in decreased smoothness of the reaching to grasping transitions. Another potential reason for the increased *T*_*pre*_ with AD would be the deficits in sensorimotor integration that is responsible for the feedback control of grasping forces according to the real-time tactile afferent information [47]. This finding would be in line with the observations from force tracking tasks that the AD patients showed prolonged reaction time and slower motion due to the deficits in precisely control the force according to visual feedback [48].

The lower *CC* and higher *TS* may suggest decreased *GF*-*LF* coordination associated with AD. During the load phase of grasping and lifting an object, the *GF* and *LF* are found to be simultaneously increased to prevent slips [49]. This *GF*-*LF* coordination is considered to be a capacity of scaling of the ratio between *GF* and *LF*, reflecting the consistency between the internal representation for the digit force prediction and the external adaptation of digit forces according to the tactile feedbacks [50]. Previous studies have found that *GF* and *LF* are related to the activation of the right intraparietal cortex, revealing the involvement of the premotor and posterior parietal cortex in *GF*-*LF* coordination during precision grip [51]. Loss of synaptic contacts and neuronal cell apoptosis in the premotor, posterior parietal cortex associated with AD therefore may lead to the compromised *GF*-*LF* coordination for precision grip.

Results further showed that the patients with AD exhibited non-bell-shaped force-rate profiles with higher *RMSE* compared with the CN during precision grip. Previous studies from arm motion [52] and isometric force production [53] found that bell-shaped force-rate profiles would be related to feedforward control strategy, whereas non-bell-shaped force-rate profiles indicate feedback-driven correction. *GFR* peak has been found to be scaled to object mass and occurs before subjects can sense the object’s mass, indicating subjects’ predictions for the object’s weight [54] or sensorimotor memory about the knowledge of the object’s physical properties (e.g. weight or mass distribution) through previous manipulations [55]. Patients with AD may thus exhibit more feedback-driven force corrections instead of feedforward control, implying potential degradation of their sensorimotor memory and motor planning.

### Effects of visual feedback on reach-to-grasp performance

Visual and somatosensory feedback is processed and integrated with motor commands and guarantees successful reach-to-grasp movements [52]. The current study observed altered reach-to-grasp kinematic parameters under different visual conditions. For example, both the AD and CN groups showed increased grasping time and minimum-jerk trajectory after the removal of visual feedback, suggesting slower motion speed and increased motion variability without visual guidance of the grasping hand. In addition, according to the results of mean absolute error, the AD groups showed more deteriorated grasping accuracy compared to the CN group after removing the visual feedback for the grasping hand and arm, suggesting that the patients with AD may suffer from lack of proprioception, relying more on the visual correction for locating their grasping hands relative to the target and for planning and executing goal-directed movements.

Visual condition could also affect the kinetic parameters of reach-to-grasp performance. The effects of visual condition were more significant in AD than in CN. Patients with AD showed much higher values of *T*_*load*_, *T*_*pre*_, CC, COP area and RMSE, and much lower values of R(b); by contrast, the CN group showed significant differences between the visual and non-visual conditions only in *T*_*pre*_. Previous studies found that visual feedback of hand and object motion contributes to estimation of digit forces and the coordination between *GF* and *LF* [56]. Consistent with these findings, the current study further revealed that the effects of AD could more obviously exhibited without visual feedback, suggesting a more reliance on visual information when controlling and coordinating kinetic parameters for reach-to-grasping performance. In addition, this study found that the AD and CN exhibited non-bell-shaped fore-rate profiles with NVF. The digit force under different visual conditions is possibly due to a higher level sensory-based control in the CNS that supports the spatiotemporal coordination of both digit forces. With VF, the central processes integrate visual, tactile, and proprioceptive information into a close-loop feedback control. This feedback control allows the two-digit motor system to coordinate flexibly in order to minimize the overall error of the force output. By contrast, the withdrawal of visual information may transfer the feedforward control mechanism to somatosensory feedback dominated by tactile and proprioceptive information. This study thus confirmed that visual feedback plays a role in feedforward and feedback control of precision grip and that the AD subjects may rely more on somatosensory feedback for force and torque control with NVF.

Correlation analyses between the reach-to-grasp parameters and the neuropsychological testing confirmed that the kinematic and kinetic changes in early-stage AD could be attributed to the degradation of neural function. With VF, the grasping time was negatively correlated with MMSE and MoCA, indicating the reduced motion speed may reflect the decline of cognitive function in the early-stage AD. Trajectory was negatively correlated with the MMSE, suggesting that the early-stage AD patients with reduced cognitive status may have difficulties in planning of motion trajectories. The *CC* was positively correlated with MMSE and MoCA, revealing that the cognitive impairment could significantly affect *GF*-*LF* coordination during precision grip due to the central or sensory dysfunction. It is noteworthy that the significant correlations between the reach-to-grasp parameters and neuropsychological assessments were highly relied on the visual feedback, and much more significant correlations were found with NVF than with VF conditions. Specially, with NVF the mean absolute error was negatively correlated with the MMSE and MoCA, and the *TS* was negatively correlated with the MMSE, which indicates that more compromised cognitive status of AD could be associated with reduced grasping accuracy and disturbed *GF*-*LF* coordination. The *T*_*pre*_ was negatively correlated with HAMA and HAMD, revealing the prolonged preload duration in AD could reflect the cognitive deficits in executive function. These results support the hypothesis that the abnormal kinematic (e.g. accuracy and coordination of reaching) and kinetic (e.g. force and moment control) characteristics would correlate with the neuropsychological status of early-stage AD, and that the reach-to-grasp kinematic and kinetic maneuver could potentially serve as a novel tool for non-invasive screening or evaluation of early-stage AD.

This study may have important implications for clinical assessment of AD. Results of this study provide evidence showing that the sensorimotor deficits are associated with AD even at the early stage. The reach-to-grasp kinematics and kinetics presented in this study may provide a basis to assess the severity and specific nature of AD on a functional level. Compared with the spatial–temporal gait measures that were recommended for evaluation of the risk of AD [57], the reach-to-grasp kinematic and kinetic measures may have higher quantification accuracy but with smaller testing space. Compared with the neuropsychological tests, braining imaging, and amyloid-beta and tau in cerebrospinal fluid that are widely used in clinical AD examination [2, 25], reach-to-grasp are noninvasive, relative low cost and easy to perform, thereby would be suitable for routine detection of AD in a large population. Future work may be performed to better identify the underlying mechanism of CNS resulting the AD-associated changes in reach-to-grasp kinematics and kinetics. The experimental set-up and test protocol presented in this study should be optimized before clinical application. For example, complex motion capture system could be replaced by more portable equipment such as wearable sensors, data gloves or leap motion cameras. In addition, more future studies are needed to demonstrate the sensitivity, specificity and reliability in assessment of the early-stage AD with this new approach.

## Conclusion

This study investigated the effects of early-stage AD on reach-to-grasp kinematics and kinetics with or without visual feedbacks. Results showed that early-stage AD could remarkably decrease the reaching speed and grasping accuracy and increase the transportation variability, extend the preload duration, disturb the *GF*-*LF* coordination, and increase the feedforward proportion in the grasping force control. The AD-related changes in the grasping kinematic and kinetic parameters were dependent on visual feedback conditions, which could be demonstrated from moderate to strong correlations of the reach-to-grasp parameters with the MMSE, MoCA, HAMA or HAMD of AD [32]. This study suggested that the early-stage AD could affect the kinematic and kinetic performance particularly without visual feedback on the grasping hand and forearm, and that the abnormal kinematic and kinetic characteristics could correlate with the status of early-stage AD. This study shed light on the effects of early-stage AD on fine motor control during reach-to-grasp behavior and may provide a novel approach to the non-invasive screening or evaluation of AD.

## Data Availability

The datasets used and/or analyzed during the current study are available from the corresponding author on reasonable request.

## References

[1] Vogel JW, Young AL, Oxtoby NP, Alexander DC (2021). Four distinct trajectories of tau deposition identified in Alzheimer’s disease. Nat Med.

[2] Scheltens P, De Strooper B, Kivipelto M, Holstege H, Chetelat G, Teunissen CE (2021). Alzheimer's disease. Lancet.

[3] Verheij S, Muilwijk D, Pel JJM, Cammen TJMVD, Steen JVD (2012). Visuomotor impairment in early-stage Alzheimer's disease: changes in relative timing of eye and hand movements. J Alzheimer's Dis..

[4] Petrella JR, Coleman RE, Doraiswamy PM (2003). Neuroimaging and early diagnosis of Alzheimer disease: a look to the future. Radiology.

[5] Arevalo-Rodriguez I, Smailagic N, Roqué I, Ciapponi A, Sanchez-Perez E, Giannakou A (2015). Mini-Mental State Examination (MMSE) for the detection of Alzheimer's disease and other dementias in people with mild cognitive impairment (MCI). Cochrane Database Syst Rev.

[6] Ganzer S, Arlt S, Schoder V, Buhmann C, Mandelkow EM, Finckh U (2003). CSF-tau, CSF-Aß1-42, ApoE-genotype and clinical parameters in the diagnosis of Alzheimer’s disease: combination of CSF-tau and MMSE yields highest sensitivity and specificity. J Neural Transm.

[7] Bahureksa L, Najafi B, Saleh A, Sabbagh M, Schwenk M (2016). The impact of mild cognitive impairment on gait and balance: a systematic review and meta-analysis of studies using instrumented assessment. Gerontology.

[8] Franssen EH, Somen L, Torossian CL, Reisberg B (1999). Equilibrium and limb coordination in mild cognitive impairment and mild Alzheimer's disease. J Am Geriatr Soc.

[9] Carment L, Abdellatif A, Lafuente-Lafuente C, Pariel S, Maier MA, Belmin J (2018). Manual dexterity and aging: a pilot study disentangling sensorimotor from cognitive decline. Front Neurol.

[10] Napier JR (1960). Studies of the hands of living primates. J Zool.

[11] Cluff T, Scott SH (2015). Apparent and actual trajectory control depend on the behavioral context in upper limb motor tasks. J Neurosci.

[12] Nashed JY, Crevecoeur F, Scott SH (2012). Influence of the behavioral goal and environmental obstacles on rapid feedback responses. J Neurophysiol.

[13] Brouwer AJD, Jarvis T, Gallivan JP, Flanagan JR (2017). Parallel specification of visuomotor feedback gains during bimanual reaching to independent goals. eNeuro..

[14] Diamond JS, Nashed JY, Johansson RS, Wolpert DM, Flanagan JR (2015). Rapid visuomotor corrective responses during transport of hand-held objects incorporate novel object dynamics. J Neurosci.

[15] Rossetti Y, Desmurget M, Prablanc C (1995). Vectorial coding of movement: vision, proprioception, or both?. J Neurophysiol.

[16] Paulignan Y, MacKenzie C, Marteniuk R, Jeannerod M (1991). Selective perturbation of visual input during prehension movements. 1. The effects of changing object position. Exp Brain Res.

[17] Jeannerod M (1984). The timing of natural prehension movements. J Mot Behav.

[18] Sober SJ, Sabes PN (2003). Multisensory integration during motor planning. J Neurosci.

[19] Johansson RS, Pruszynski JA, Flanagan JR (2016). A rapid tactile-motor reflex automatically guides reaching toward handheld objects. Curr Biol.

[20] Johansson RS, Flanagan JR (2009). Coding and use of tactile signals from the fingertips in object manipulation tasks. Nat Rev Neurosci.

[21] Li K, Li ZM (2013). Cross recurrence quantification analysis of precision grip following peripheral median nerve block. J Neuroeng Rehabil.

[22] Hu W, Wei N, Li ZM, Li K (2018). Effects of muscle fatigue on directional coordination of fingertip forces during precision grip. PLoS ONE.

[23] Nataraj R, Pasluosta C, Li ZM (2014). Online kinematic regulation by visual feedback for grasp versus transport during reach-to-pinch. Hum Mov Sci.

[24] Lemon RN, Johansson RS, Westling G (1995). Corticospinal control during reach, grasp, and precision lift in man. J Neurosci.

[25] Montero-Odasso M, Ismail Z, Camicioli R (2020). Alzheimer disease, biomarkers, and clinical symptoms-quo vadis?. JAMA Neurol.

[26] Shen ZL, Mondello TA, Nataraj R, Domalain MF, Li ZM (2012). A digit alignment device for kinematic analysis of the thumb and index finger. Gait Posture.

[27] Nataraj R, Li ZM (2013). Robust identification of three-dimensional thumb and index finger kinematics with a minimal set of markers. J Biomech Eng.

[28] Nataraj R, Evans PJ, Seitz WH, Li ZM (2014). Pathokinematics of precision pinch movement associated with carpal tunnel syndrome. J Orthop Res.

[29] Flash T, Hogan H. The coordination of arm movements : an experimentally confirmed mathematical model. J Neuroscience. 1985;5(7):1688-703. 10.1523/JNEUROSCI.05-07-01688.1985 PMC6565116 4020415

[30] Dun S, Kaufmann RA, Li ZM (2007). Lower median nerve block impairs precision grip. J Electromyogr Kinesiol.

[31] Flanagan JR, Burstedt MK, Johansson RS (1999). Control of fingertip forces in multidigit manipulation. J Neurophysiol.

[32] Swinscow TDV, Campell MJ. Statistics at Square One. 9th edn, Plymouth Latimer Trend Company Ltd.; 1996.

[33] Waite LM, Broe GA, Grayson DA, Creasey H (2000). Motor function and disability in the dementias. Int J Geriatr.

[34] Benedict RHB, Holtzer R, Motl RW, Foley FW, Kaur S, Hojnacki D (2011). Upper and lower extremity motor function and cognitive impairment in multiple sclerosis. J Int Neuropsychol Soc.

[35] Goldman WP, Baty JD, Buckles VD, Sahrmann S, Morris JC (1999). Motor dysfunction in mildly demented AD individuals without extrapyramidal signs. Neurology.

[36] Aggarwal NT, Wilson RS, Beck TL, Bienias JL, Bennett DA (2006). Motor dysfunction in mild cognitive impairment and the risk of incident Alzheimer disease. Arch Neurol.

[37] Agosta F, Rocca MA, Pagani E, Absinta M, Magnani G, Marcone A (2010). Sensorimotor network rewiring in mild cognitive impairment and Alzheimer's disease. Hum Brain Mapp.

[38] Yan JH, Rountree S, Massman P, Doody RS, Li H (2008). Alzheimer’s disease and mild cognitive impairment deteriorate fine movement control. J Psychiatr Res.

[39] Julia S, Peraza LR, Michael F, Thomas AJ, Marcus K, Peter G (2019). Dysfunctional brain dynamics and their origin in Lewy body dementia. Brain.

[40] Hikosaka O, Nakamura K, Sakai K, Nakahara H (2002). Central mechanisms of motor skill learning. Curr Opin Neurobiol.

[41] Jeannerod M, Arbib MA, Rizzolatti G, Sakata H (1995). Grasping objects: the cortical mechanisms of visuomotor transformation. Trends Neurosci.

[42] Halsband U, Lange R (2006). Motor learning in man: a review of functional and clinical studies. J Physiol Paris.

[43] Thiyagesh SN, Farrow TD, Parks RW, Accosta-Mesa H, Young C, Wilkinson ID (2009). The neural basis of visuospatial perception in Alzheimer's disease and healthy elderly comparison subjects: an fMRI study. Psychiatry Res Neuroimaging.

[44] Knierim JJ, Neunuebel JP, Deshmukh SS (2014). Functional correlates of the lateral and medial entorhinal cortex: objects, path integration and local–global reference frames. Philos trans R Soc Lond.

[45] Howett D, Castegnaro A, Krzywicka K, Hagman J, Marchment D, Henson R (2019). Differentiation of mild cognitive impairment using an entorhinal cortex-based test of virtual reality navigation. Brain.

[46] Gulde P, Leippold K, Kohl S, Grimmer T, Diehl-Schmid J, Armstrong A (2018). Step by step: kinematics of the reciprocal trail making task predict slowness of activities of daily living performance in Alzheimer’s disease. Front Neurol.

[47] Fellows S, Schwarz M, Schaffrath C, Dömges F, Noth J (1997). Disturbances of precision grip in Huntington's disease. Neurosci Lett.

[48] Bartoli E, Caso F, Magnani G, Baud-Bovy G (2017). Low-cost robotic assessment of visuo-motor deficits in Alzheimer's disease. IEEE Trans Neural Syst Rehabil Eng.

[49] Hu WJ, Wei N, Li ZM (2018). Effects of muscle fatigue on directional coordination of fingertip forces during precision grip. PLoS ONE.

[50] Li K, Wei N, Cheng M, Hou X, Song J (2018). Dynamical coordination of hand intrinsic muscles for precision grip in diabetes mellitus. Sci Rep.

[51] Ehrsson HH, Fagergren A, Johansson RS, Forssberg H (2003). Evidence for the involvement of the posterior parietal cortex in coordination of fingertip forces for grasp stability in manipulation. J Neurophysiol.

[52] Gordon J, Ghilardi MF, Ghez C (1995). Impairments of reaching movements in patients without proprioception. II. Effects of visual information on accuracy. J Neurophysiol..

[53] Vicario DS, Ghez C (1984). The control of rapid limb movement in the cat. IV. Updating of ongoing isometric responses. Exp Brain Res.

[54] Johansson RS, Westling G (1988). Coordinated isometric muscle commands adequately and erroneously programmed for the weight during lifting task with precision grip. Exp Brain Res.

[55] Green S, Grierson L, Dubrowski A, Carnahan H (2010). Motor adaptation and manual transfer: insight into the persistent nature of sensorimotor representations. Brain Cogn.

[56] Sarlegna FR, Baud-Bovy G, Danion F (2010). Delayed visual feedback affects both manual tracking and grip force control when transporting a handheld object. J Neurophysiol.

[57] Bennett DA, Schneider JA, Buchman AS, Barnes LL, Boyle PA, Wilson RS (2012). Overview and findings from the rush Memory and Aging Project. Curr Alzheimer Res.

